# Prevalence and Detection of *qac* Genes from Disinfectant-Resistant *Staphylococcus aureus* Isolated from Salon Tools in Ishaka Town, Bushenyi District of Uganda

**DOI:** 10.1155/2020/1470915

**Published:** 2020-08-12

**Authors:** Solange Gahongayire, Adamu Almustapha Aliero, Charles Drago Kato, Alice Namatovu

**Affiliations:** ^1^Department of Microbiology and Immunology, Faculty of Biomedical Sciences, Kampala International University, Western Campus, P.O. Box 71, Bushenyi, Kampala, Uganda; ^2^School of Biosecurity, Biotechnical and Laboratory Sciences, College of Veterinary Medicine, Animal Resources and Biosecurity, Makerere University, P.O. Box 7062, Kampala, Uganda; ^3^Department of Biotechnical and Diagnostic Sciences, College of Veterinary Medicine, Animal Resources and Biosecurity, Makerere University, P. O. Box 7062, Kampala, Uganda

## Abstract

Bacterial infections are on a rise with causal-resistant strains increasing the economic burden to both patients and healthcare providers. Salons are recently reported as one of the sources for transmission of such resistant bacterial strains. The current study aimed at the identification of the prevalent bacteria and characterization of quaternary ammonium compound (*qac*) genes from disinfectant-resistant *S. aureus* isolated from salon tools in Ishaka town, Bushenyi District of Uganda. A total of 125 swabs were collected from different salon tools (combs, brushes, scissors, clippers, and shaving machines), and prevalent bacteria were isolated using standard microbiological methods. Identification of isolated bacteria was done using standard phenotypic methods including analytical profile index (API). Susceptibility patterns of the isolated bacteria to disinfectant were determined using the agar well diffusion method. Quaternary ammonium compound (*qac*) genes *(qac*A/B and *qac*C) associated with disinfectant resistances were detected from disinfectant-resistant *S. aureus* using multiplex polymerase chain reaction (PCR) and Sanger sequencing methods. Of the 125 swab samples collected from salons, 78 (62.4%) were contaminated with different bacteria species. Among the salon tools, clippers had the highest contamination of 20 (80.0%), while shaving machines had the lowest contamination of 11 (44.0%). The most prevalent bacteria identified were *Staphylococcus epidermidis* (28.1%) followed by *S. aureus* (26.5%). Of all the disinfectants tested, the highest resistance was shown with sodium hypochlorite 1%. Out of the eight (8) disinfectant-resistant *S. aureus* analysed for *qac* genes, 2 (25%) isolates (STP6 and STP9) were found to be *qac*A/B positive, while 2 (25%) isolates (STP8 and STP9) were found to be *qac*C gene positive. This study has shown that bacterial contamination of salon tools is common, coupled with resistance to disinfectants with sodium hypochlorite resistance being more common. Furthermore, observed resistance was attributed to the presence of *qac* genes among *S. aureus* isolates. A search for *qac* genes for disinfectant resistance from other bacteria species is recommended.

## 1. Introduction

Worldwide, skin infections are among the important causes of morbidity [1–3]. Among these infections are those caused by different bacteria which are common in most developing countries [4]. These infections can be spread through different ways including direct contact with bodily fluids from contaminated blood, pus, sores, cuts, or grazes [5, 6]. Beauty salons (defined as places where hair, face, and body can be given special treatments to improve their appearance) pose potential health risks to clients and service providers in terms of skin infections and sometimes physical injuries [7, 8]. Therefore, bacterial skin infections can be acquired from different inanimate objects depending on the nature of the service sought, tools and equipment used, the health status of the clients and service providers, as well as the infection control procedures implemented [5].

Several bacteria, viruses, and fungi especially yeasts have been isolated from manicure, pedicure, hairdressing, and barbering equipment [8–12]. Studies done in the United States of America on manicure and pedicure showed evidence of *Streptococcus* spp., *Enterococcus* spp., *Micrococcus* spp., *Bacillus* spp., *Enterobacter* spp., *Klebsiella* spp., *Acinetobacter* spp., *Citrobacter* spp., and *Escherichia coli* [11, 13]. Other studies from California [12], two urban and two semiurban areas in South Africa [14], Cincinnati [15], and North Carolina counties, USA [16], on some tools also isolated other bacteria such as *Mycobacterium fortuitum*, *M. chelonae*, and *M. mageritense*. Similarly, studies from Abia state, Nigeria [8], Adamawa state, Nigeria [17], and Pakistan [10] based on in-use tools in hairdressing and barbering showed contamination by bacteria like *S. aureus*, *Pseudomonas aeruginosa*, *S. epidermidis*, *Streptococcus* spp., *Enterococcus* spp., and *Enterobacter* spp. However, fewer studies have investigated the role of salons in the spread of drug-resistant pathogens, especially in Africa.

Proper use of disinfectants can help contain and prevent the spread of these pathogens from inanimate objects. However, the study has revealed resistance of some microorganisms on a different type of disinfectants [18]. A study done by Guimarães et al. [19] on different disinfectants revealed that bacteria resistance to phenols and quaternary ammonium compound (*qac*) was common. Among these resistant strains were methicillin-resistant *S. aureus* (MRSA), *S. epidermidis*, *Enterobacter cloacae, Proteus mirabilis*, *Serratia marcescens*, and *P. aeruginosa*. Several factors such as concentration used, application method, the contact time of the disinfectant, and the safety considerations for operators to apply disinfectants may affect the effectiveness of disinfectants [20–22]. Uganda National Bureau of Standard (UNBS) [23] gives guidelines pertaining to the usage of disinfectants; however, it is not clear if they are followed by salons in Uganda. Furthermore, UNBS recommends testing for effectiveness of disinfectants, but there is a paucity of such studies in Uganda, and the role of the salon in the spread of infections in Uganda has not yet been elucidated. This study, therefore, explored the effectiveness of the commonly used disinfectants and identified the genes associated with disinfectant resistance in some of the resistant strains obtained from salon tools in Bushenyi district, Uganda.

## 2. Materials and Methods

### 2.1. Study Design

The cross-sectional study was carried out on selected beauty salons in Ishaka town located in Bushenyi district of Uganda. Different salons barbershops, hairdressing/ladies salons, and unisex salons were selected purposively based on salons who claim to use disinfectants and others methods to sterilize their materials, while the choice of selected tools was in accordance with literature indicating that the tools selected for this study are the most commonly contaminated [9].

### 2.2. Sample Collection

One hundred and twenty-five (125) swab samples collected from different salons barbershops, hairdressing/ladies salons, and unisex salons. Twenty-five (25) samples each were collected from different salon tools such as combs, brushes, scissors, clippers, and shaving machines using moistened swabs with 0.85% normal saline according to the method described by Enemuor et al. [9]. Swabs were later inserted in sterile 5 ml tubes containing 2 ml of Stuart transport medium and transported in ice box cooler to the Microbiology Laboratory, Kampala International University Western Campus for further analysis.

### 2.3. Isolation and Identification of Bacteria

Isolation and identification of bacteria were done using standard microbiological protocols. Primary isolation of bacteria was done by inoculating the swabs on nutrient agar plates and then incubated at 37°C for 18–24 hours. The suspected bacteria were subcultured on blood agar plates and incubated at 37°C for 18–24 hours. Later colonies examined for hemolytic characteristics (alpha, beta, or gamma). Furthermore, bacterial isolates were confirmed to species level using colony morphology from different media (including nutrient agar, blood agar, Mannitol salt agar, and MacConkey agar), Grams staining, and biochemical tests. Deferoxamine susceptibility test was done to differentiate between *S. aureus* and other *Staphylococcus* spp. where *S. hominis* and *S. epidermidis* are sensitive, while *S. aureus* is resistant. Fosfomycin susceptibility test was also carried out to confirm *S. epidermidis* isolate [24]. *Staphylococcus* spp. were further confirmed using analytical profile index (API) system [25].

### 2.4. Disinfectants Susceptibility Test

Disinfectants susceptibility tests were done using the agar well diffusion method as described by Günther et al. [26] with some modifications. The bacterial inocula were standardized with 0.5 McFarland standard solutions. This was inoculated on freshly prepared Mueller–Hinton agar (MHA) using sterile swabs after which wells were made using sterile cork borer (6 mm). The concentration of different disinfectant was prepared: methylated spirit (70%), sodium hypochlorite (1%), and surgical spirit BP (70%). Amoxicillin 0.1 mg/ml and sterile distilled water were used as positive and negative controls, respectively. A hundred microliters (100 *μ*l) of each disinfectant, positive and negative controls, were pipetted using a sterile pipette (100–1000 *μ*l Eppendorf™, Reference™, Fisher Scientific) and filled into the well and allowed to stand for 10 minutes after which the plates were incubated at 37°C for 18–24 hr [27]. Inhibition zones were observed after the incubation period and were interpreted based on the absence or presence of inhibition zone and interpreted according to International Clinical Laboratory Standard guidelines [28].

### 2.5. Molecular Detection of *qac* Genes

#### 2.5.1. DNA Extraction

Genomic DNA extraction was done on the eight *S. aureus* isolates that showed resistance to all disinfectants. Extraction of DNA was done according to the method previously described [29] with some slight modifications. In brief, 500 *μ*l of the 24 hr culture was mixed with 500 *μ*l of reagent grade water and heated at 100°C for 10 minutes and later centrifuged at 15000 rpm for 15 min. The supernatant containing genomic DNA was then conserved in −20°C until use for polymerase chain reaction (PCR) amplification experiments.

#### 2.5.2. PCR Amplification and Sequencing

The primers sequences used were as follows: *qac*A/B: forward primer 5′-CTAT GGCAATAGG AGA TATGGTGT, reverse primer 5′-CCACTACAGATTCTTCAGCTACATG-3′; *qac*C: forward primer5′-AAACAATGCAACACCTACCACT, reverse primer 5′-AACG AAACTA CGCC GACTATG-3′ [30]. PCR was performed in 25 *μ*l final reaction volume containing 6 *μ*l (50 ng) of bacterial extracted DNA from *S. aureus*-resistant species and then added 2 *μ*l of forward primer (10 pmol), 2 *μ*l of reverse primer (10 pmol), Taq 12.5 *μ*l master mix solution, and 2.5 *μ*l of nuclease-free water. Amplification was done in Thermal Cycler (Model 480, Perkin-Elmer Cetus, Foster City, CA, USA). The PCR was performed under the following conditions: initial denaturation at 95°C for 3 min, then 35 cycles of three-step PCR amplification consisting of denaturation at 94°C for 1 min, primer annealing at 55°C for 30 seconds, and extension at 72°C for 2 min, and final extension was attained for 10 min [29, 30]. Following PCR amplification, the product was digested with 5U of restriction enzyme (Alu-I) at 37°C for 90 min and after the product was analysed using electrophoresis on a 2% (w/v) agar rose/1 × TBE buffer. The expected sizes of amplified fragments of *qac*B, *qac*A, and *qac*C genes were 176 bp, 220 bp, and 149, respectively [30]. The amplified products were photographed, and their size was determined using a 25 bp molecular size marker (Promega, USA). After gel electrophoresis, the positive isolates were further confirmed by sequencing using the Sanger sequencing method as described by Xiao et al. [31]. All PCR and sequencing work were done at Inqaba biotechnology laboratory company, South Africa.

### 2.6. Data Analysis

Data were entered in MS excel after being edited and cleaned of any obvious errors. The data were then analysed using the Statistical Package for Social Sciences (SPSS), version 21 software. Data on prevalent bacteria on the contaminated beauty salon tools were presented in the form of percentages, comparison among salon tools was done using a chi-square test, and *p* value ≤0.05 was considered significant. Disinfectant-resistant genes (*qac*) identified from *Staphylococcus* species were analysed using BLAST search (https://blast.ncbi.nlm.nih.gov/Blast.cgi) in order to obtain the phylogenetic tree. The obtained sequences (of related *Staphylococcus* species with *qac* genes from NCBI) and our sequences were aligned using the MUSCLE alignment method, and a phylogenetic tree was constructed using join maximum likelihood using 1000 bootstrap replica. All sequences were analysed using MEGA software version 6.0. The constructed phylogenetic tree was interpreted accordingly especially the formation clade of our isolates and other known isolates obtained from NCBI. A clade consists of an organism and all of its descendants.

### 2.7. Ethical Considerations

Ethical clearance was sought from the Research Ethics Committee of Kampala International University Western Campus (KIU-WC) (Ref: SF201813). Salon owners' approval was obtained to collect samples from different tools.

## 3. Results

### 3.1. Prevalent Bacteria Isolated from Beauty Salon Tools

Out of 125 salons tools swabbed, 78 (62.4%) were contaminated with a total of 196 different bacterial isolates identified. On comparison of microbial load across the different saloon tools, clippers were found to have a higher contamination among the salon tools studied (80.0%), followed by brushes (76.0%), scissors (64.0%), combs (48.0%), and shaving machine (44.0%) (Table 1). The most prevalent bacteria identified were *S. epidermidis* (28.1%) and *S. aureus* (26.5%). Other bacterial species involved in the contamination of salon tools were *Streptococcus* spp. (16.8%), *Micrococcus* spp. (12.8%), *S. xylosus* (8.8%), *P. aeruginosa (*8.0%), *Enterobacter aerogenes* (6.4%), *Serratia liquefaciens* (6.4%), *Bacillus subtilis* (4.8%), *Bacillus* spp. (4.0%), *Serratia marcescens* (1.6%), *Enterobacter* spp. (0.8%), *and Staphylococcus sciuri* (0.8%), as indicated in Table 2.

The distribution of bacterial contaminant according to the type of salons showed that barbershops and unisex salons had the highest distribution with (37%) each, while hairdressing/ladies salons had the least (27.0%) (Table 3).

### 3.2. Susceptibility Patterns of Isolated Bacteria to Commonly Used Disinfectants

Out of the 137 bacterial isolates subjected to disinfectant susceptibility tests, 37 (27.0%) were resistant to 1% sodium hypochlorite and 22 (16.0%) to 70% surgical spirit BP, while 4 (2.9%) were resistant to methylated spirit (70%). It was observed that some isolates of *S. epidermidis*, *S. xylosus*, and *E. aerogenes* were resistant to both surgical spirit BP (70%) and sodium hypochlorite (1%). However, *S. marcescens* isolates were found to be resistant to both methylated spirit (70%) and sodium hypochlorite (1%), while isolates of *S. aureus* were found to be resistant to all three disinfectants, i.e., surgical spirit BP (70%), sodium hypochlorite (1%), and methylated spirit (70%).

The resistant pattern of bacterial isolates to disinfectants varies. *S. liquefaciens* isolates showed 87% resistant to surgical spirit BP (70%), *S. marcescens* showed 50% resistant to methylated spirit (70%), while *S. liquefaciens, S. marcescens*, and *S. sciuri* showed 100% resistant to sodium hypochlorite (1%). The distribution of resistance according to the bacterial species showed that *S. liquefaciens, S. marcescens*, and *S. sciuri* were 100% resistant to sodium hypochlorite, while *S. epidermidis* showed low resistance (3.6%). Similarly, *S. liquefaciens* showed high resistance (87.5%) to 70% surgical spirit BP among the bacteria tested, while *S. marcescens* and *S. sciuri* showed no resistance (0.0%) to this disinfectant. *S. marcescens* was found to be the most resistant bacterial to methylated spirit, while *S. epidermidis, S. xylosus, E. aerogenes*, and *S. sciuri* showed no resistance (0.0%) (Table 4).

The results on the distribution of resistant bacterial isolates according to salon type showed that barbershops had the highest distribution of resistant bacterial isolates 20 (41.7%), while hairdressing/ladies salons had the lowest resistant bacterial isolates 5 (14.3%). From the barbershop salons, it was observed that *S. liquefaciens, E. aerogenes*, and *S. liquefaciens* showed 100% resistance to the disinfectant, while *S. sciuri* had no resistance to the disinfectants tested. However, it was also observed that *S. liquefaciens* from hairdressing/ladies salons showed 100% resistance to the disinfectants tested, while *S. epidermidis, S. xylosus, E. aerogenes, S. marcescens*, and *S. sciuri* showed no resistance to the disinfectant tested. In unisex salons, *S. liquefaciens, E. aerogenes*, and *S. sciuri* showed 100% resistance to disinfectants tested, while *S. marcescens* showed no resistance (Table 5).

### 3.3. Characterization of *qac* Genes from *S. aureus*

Eight isolates of *S. aureus* that showed resistance to all the three disinfectants were subjected to Multiplex PCR for detection of *qac*A/B and *qac*C genes associated with disinfectant resistance. Approximately, 220 base pair (bp) and 249 base pair (bp) amplicon size were obtained for *qac*A and *qac*C genes, respectively. Out of the eight (8) bacterial isolates that were analysed for *qac* genes, 2 (25%) isolates (STP6 and STP9) were found to be *qac*A gene positive, while 2 (25%) isolates (STP8 and STP9) were found to be *qac*C gene positive.

**Figure 1:**
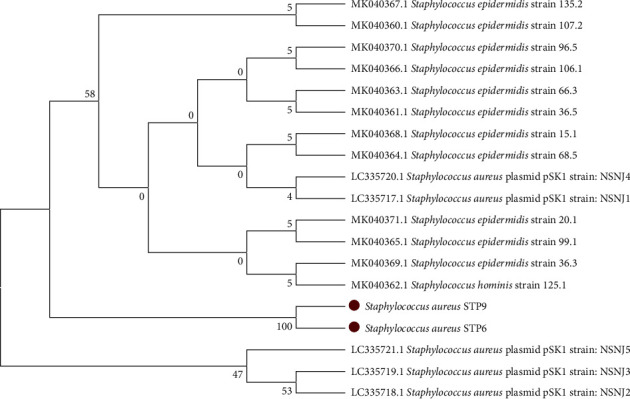
Phylogenetic tree constructed for 17 different *qac*A proteins extracted from GenBank, together with two *S. aureus* isolates (STP6 and STP9) that were *qac*A gene positive in this study. The tree was constructed using MEGA software (version 6) using maximum likelihood at 1000 replica.

Sequence analysis results for both isolates showed that the antiseptic resistance protein (*qac*A) gene sequence of the two *S. aureus* was 100% similar to about 17 *Staphylococcus* spp. (Table 6). A phylogenetic tree was constructed using the maximum likelihood statistical method at 1000 number of bootstrap replication results from the phylogenetic tree did not yield any significant bootstrap values (Figure 1).

## 4. Discussion

Bacterial infections from resistant strains are on the rise causing an increased economic burden to both patients and healthcare providers [2, 3]. Salon shops have been incriminated in the spread of these bacterial pathogens including resistant strains [37, 38]. The resistance of these bacteria to disinfectant can lead to failure to fight both community- and hospital-acquired infections [39]. Detection of genes associated with disinfectant resistance is clinically important in the treatment of these infections as these genes vary and confer reduced susceptibility to commonly used antiseptics and disinfectants [40]. This study was therefore designed to determine prevalence and susceptibility of bacteria isolated from salons to commonly used disinfectants in Ishaka town, Bushenyi District of Uganda. The prevalence of bacterial contamination from studied salons in this study was lower compared to the findings of Enemuor et al. [9] who reported 100% (*n* = 42) prevalence of hairdressing and beauty salons tools contamination from Nigeria and so was the finding from a study by Dadashi and Dehghanzadeh [40] on shared cosmetic kits in women beauty salons in Iran. The lower prevalence found in this study could be due to the awareness of the majority (88%) of the salon's operators on biosafety guideline. According to Guimarães et al. [19], the higher prevalence of contamination found in their study could be due to inappropriate sterilization techniques used by these salon operators in which 38% of their studied participants used ultraviolet (UV) light, 18% used glass beads, and 1% used ultrasonic cleaners, all of which are not approved methods of sterilization in many jurisdictions [41].

The isolation of *S. epidermidis as* the most frequently isolated bacteria contaminant from salons tools was contrary to the findings of Enemuor et al. [9] and Dadashi and Dehghanzadeh [40] who reported higher distribution of *S. aureus* from hairdressing and beauty salons tools. Isolation of bacteria from studied salons tools in the current study probably indicates that the disinfectant or sterilization methods used by salon operators were not effective or failure to use disinfectants. The contamination of all salons tools with clippers having the highest contamination was in line with the study by Omoruyi and Idemudia [21], who reported prevalence of 91.67%, 75%, and 83.3% of *Staphylococcus*, *Streptococcus*, and *Bacillus* species, respectively, from barbing clippers in Benin City, Nigeria. This study finding was contrary to the findings of Stanley et al. [8], who reported a higher bacterial contamination of dryer although clippers were part of the study. Naz et al. [10] also reported 100%, 100%, and 88% of *S. aureus* contamination in sponge, brush, and wax, respectively, from cosmetic tools used in beauty salons from different areas of Lahore, Pakistan. The higher bacterial contaminations observed from clipper in this study could be due to the improper sterilization or the ineffectiveness of the disinfectant used by these salon operators although the majority of studied salons operators claimed to sterilize or disinfect the tools after use on each client.

The higher bacteria resistance to sodium hypochlorite 1% was in line with findings of Al-Jubory et al. [42] who reported resistance of *P. aeruginosa* isolated in AL-Hilla Teaching Hospital Iraq to sodium hypochlorite 6%. This was contrary to the findings of Awodele et al. [20], who also reported susceptibility of *P. aeruginosa* and *B. subtilis* isolated from Barbing salons Clipper in Benin City, Edo State, Nigeria, to sodium hypochlorite 100% concentration although the concentration was higher than that used in this study. The differences in resistance observed from these studies could be due to the difference in the concentration of sodium hypochlorite used or difference in nature of the bacterial isolates used.

The low resistance to methylated spirit was in line with findings of Awodele et al. [20] who reported susceptibility of *P. aeruginosa* and *B. subtilis* to methylated spirit with 20 and 22 mm zone of inhibition. The lower resistance of the isolated bacteria to the methylated spirit in this study could be linked to the knowledge of salon operators as in this study, and it showed that most salon operators are knowledgeable about guidelines designed by UNBS, which recommended the usage of methylated spirit at 70% as a disinfectant for basins and metal tools and as in the study by Awodele et al. [20]. Proper cleaning of salon equipment with right disinfectant concentration reduces the risk of bacteria developing resistant to disinfectants. This was demonstrated by a study by Awodele et al. [20] who reported 100% *B. subtilis* and *P. aeruginosa* resistance to methylated spirit at concentrations of 6.25%, 12.5%, and 50% although the disinfectant (methylated spirit) showed antibacterial activity at 100% concentration.

Presence of bacteria pathogens within the community is chained from animal and food industries to the community or hospitals to community [39]. These sectors use disinfectant in their day to day activities. In this study, detection of *qac* genes was limited to resistant *S. aureus* but not the other resistant strains due to lack of literature on the detection of *qac* genes in other species. The prevalence of *qac*A/B and *qac*C genes associated with disinfectant resistance reported in this study is lower than the prevalence reported by a study conducted within the community of Hong Kong, China, on an automated teller machine, in which 11% of the isolated *S. aureus* carried *qac*A/B and less than 2% had *qac*C/smr, while *qac*C/smr was found in 14% and *qac*A/B in 26% of the coagulase-negative Staphylococci (CNS) isolates [43]. This was contrary to the findings of Wong et al. [44] who reported zero prevalence of *qac*C/smr and *qac*A/B genes associated with disinfectant genes in methicillin-resistant *S. aureus* isolated in porcine although another gene associated with disinfectant resistant such as *qac*G was detected. The genes *qac*A/B and *qac*C/smr have all been detected from *E. coli* resistant to beta-lactam antibiotics in bovine, caprine, and other food-related coagulase-negative *Staphylococcus* spp. such as *S. epidermidis, S. saprophyticus*, *S. cohnii*, and *S. hominis* [39, 45, 46].

However, the presence of *qac* genes associated with disinfectant resistant was also reported in many studies involving a hospital or clinical sample [47–52]. Study on *qac* genes associated with disinfectant resistance in African countries seems to be scarce and was reported to be higher in Asia [39]. For example, Bjorland et al. [46] reported 7.5% of *qac*A/B genes in clinical isolates of 32% MRSA in Japan. Similarly, in a study conducted by Conceição et al. [51], from three African countries (Angola, São Tomé and Príncipe, and Cape Verde) among 82 methicillin-resistant *S. aureus* (MRSA) and 219 methicillin-susceptible *S. aureus* (MSSA) isolated from previous nasal carriage showed 40.5% *qac*A/B genes.

The phylogenetic tree analysis constructed using maximum likelihood method at 1000 number of bootstrap replications showed that the two *S. aureus* isolates (STP9 and STP6) form a different clade from known *Staphylococcus* spp. that harboured antiseptic resistance protein (*qac*A) gene extracted from Gene bank (NCBI). All the isolates used for comparisons were mostly isolated from clinical samples as compared to ours which were isolated from environmental sources (salons). Secondly, most of the antiseptic resistance protein (*qac*A) gene sequences, *Staphylococcus* spp. extracted from Gene bank (NCBI) were whole-genome sequences, while our isolates were partial sequences, and this could explain the reason why the two *S. aureus* isolates (STP9 and STP6) form a new clade in the tree. The results of this finding were in line with the findings of Wassenaar et al. [39], who reported variations in the phylogenetic analysis of antiseptic resistance protein (*qac*A) gene sequences of different species of Staphylococci sequences extracted from Gene bank.

## 5. Conclusion

Our study has shown that bacteria contamination on salon equipment in Bushenyi district of Uganda is common, with *S. epidermidis* commonly isolated. Most disinfectant-resistant isolates were commonly reported against sodium hypochlorite 1%. The disinfectant-resistant *S. aureus* isolated had either *qac*A or *qac*C genes. Furthermore, phylogenetic analysis of the two *Staphylococci* spp. sequences showed that they harboured *qac*A genes. Presence of disinfectant-resistant *S. aureus* harbouring *qac* genes from these studied salons showed the need for frequent proper sensitization of these salons' operators on the proper use of disinfectants by following the biosafety guideline of salon operation to cub development of disinfectant-resistant genes among the bacterial community of salons which can spread within the community. However, there is a need for further study on characterization and detection of the *qac* genes of other bacteria species isolated from salon tools from this studied area as this can help in proper diagnosis and treatments of diseases caused by these bacteria.

## Figures and Tables

**Table 1:** Contamination of beauty salon tools in Ishaka town, Uganda.

Salon tools	Number examined	Contamination		*p* value
		Positive, *n* (%)	Negative, *n* (%)	
Clippers	25	20 (80.0)	5 (20.0)	0.034^*∗*^
Brushes	25	19 (76.0)	6 (24.0)	0.04^*∗*^
Scissors	25	16 (64.0)	9 (36.0)	0.52
Combs	25	12 (48.0)	13 (52.0)	0.32
Shaving machine	25	11 (44.0)	14 (56.0)	0.43

Total	125	78 (62.4)	47 (37.6)	

^*∗*^Statistically significant at *p* ≤ 0.05.

**Table 2:** Prevalent bacteria isolated from beauty salon tools in Ishaka town, Uganda.

Organisms	Barbershops *n* (%)	Hairdressing/ladies salons *n* (%)	Unisex salons *n* (%)	Total (%)	*p* value
*S. epidermidis*	21 (10.7)	18 (9.2)	16 (8.2)	55 (28.1)	0.045^*∗*^
*S. aureus*	12 (6.1)	12 (6.1)	28 (14.3)	52 (26.5)	0.02^*∗*^
*Streptococcus* spp.	8 (4.1)	7 (3.6)	7 (3.6)	22 (11.2)	0.061
*Micrococcus* spp.	5 (2.6)	5 (2.6)	6 (3.1)	16 (8.2)	0.06
*S. xylosus*	5 (2.6)	2 (1.0)	4 (2.0)	11 (5.6)	0.53
*P. aeruginosa*	4 (2.0)	3 (1.5)	3 (1.5)	10 (5.1)	0.54
*S. liquefaciens*	3 (1.5)	3 (1.5)	2 (1.0)	8 (4.1)	0.04^*∗*^
*E. aerogenes*	5 (2.6)	0 (0.0)	3 (1.5)	8 (4.1)	0.71
*B. subtilis*	3 (1.5)	2 (1.0)	1 (0.5)	6 (3.1)	0.32
*Bacillus* spp.	4 (2.0)	0 (0.0)	1 (0.5)	5 (2.6)	0.52
*S. marcescens*	2 (1.0)	0 (0.0)	0 (0.0)	2 (1.0)	0.63
*S. sciuri*	0 (0.0)	0 (0.0)	1 (0.5)	1 (0.5)	1.0

Total	72 (37.0)	52 (27.0)	56 (37.0)	196 (100.0)	

^*∗*^Statistically significant at *p* ≤ 0.05.

**Table 3:** Prevalent bacteria isolated from beauty salon in Ishaka town, Uganda, according salon tool.

Organisms	Comb (%)	Brush (%)	Clipper (%)	Scissor (%)	Shaving machine (%)	Total (%)	*p* value
*S. epidermidis*	8 (14.5)	14 (25.5)	18 (32.7)	9 (16.4)	6 (10.9)	55 (28.1)	0.70
*S. aureus*	9 (17.6)	12 (23.5)	14 (27.5)	7 (11.8)	10 (19.6)	52 (26.5)	0.67
*Streptococcus* spp.	0 (0.0)	7 (33.3)	6 (28.6)	6 (28.6)	3 (14.3)	22 (11.2)	0.057^*∗*^
*Micrococcus* spp.	2 (12.5)	2 (12.5)	4 (25.0)	4 (25.0)	4 (25.0)	16 (8.2)	0.56
*S. xylosus*	4 (36.4)	2 (18.2)	0 (0.0)	2 (18.2)	3 (27.3)	11 (5.6)	0.04^*∗*^
*P. aeruginosa*	3 (30.0)	2 (20.0)	2 (20.0)	1 (10.0)	2 (20.0)	10 (5.1)	0.46
*S. liquefaciens*	5 (62.5)	0 (0.0)	1 (12.5)	1 (12.5)	1 (12.5)	8 (4.1)	0.002^*∗*^
*E. aerogenes*	0 (0.0)	2 (25.0)	1 (12.5)	3 (37.5)	2 (25.0)	8 (4.0)	0.34
*B. subtilis*	0 (0.0)	3 (50.0)	1 (16.7)	2 (33.3)	0 (0.0)	6 (3.1)	0.03^*∗*^
*Bacillus* spp.	1 (20.0)	1 (20.0)	2 (40.0)	1 (20.0)	0 (0.0)	5 (2.6)	0.068
*S. marcescens*	0 (0.0)	0 (0.0)	2 (100.0)	0 (0.0)	0 (0.0)	2 (1.0)	—
S. sciuri	0 (0.0)	0 (0.0)	0 (0.0)	1 (100.0)	0 (0.0)	1 (0.5)	—

^*∗*^Statistically significant at *p* value ≤0.05.

**Table 4:** Disinfectant-resistant bacterial isolates in Ishaka town, Uganda.

Organisms	No. examined	Types of disinfectants tested		
		Surgical spirit BP (70%) Resistant strains (%)	Methylated spirit (70%) Resistant strains (%)	Sodium hypochlorite (1%) Resistant strains (%)
*S. epidermidis*	55	2 (3.6)	0 (0.0)	2 (3.6)
*S. aureus*	52	8 (15.3)	2 (3.8)	9 (17.3)
*S. xylosus*	11	3 (27.2)	0 (0.0)	8 (72.7)
*S. liquefaciens*	8	7 (87.5)	1 (12.5)	8 (100.0)
*E. aerogenes*	8	2 (25.0)	0 (0.0)	7 (87.5)
*S. marcescens*	2	0 (0.0)	1 (50.0)	2 (100.0)
*S. sciuri*	1	0 (0.0)	0 (0.0)	1 (100.0)

Total	137	22 (16.1)	4 (2.9)	37 (27.0)

**Table 5:** Distribution of resistant bacteria isolates based on type of salons from Ishaka town, Uganda.

Organisms	Types of salons					
	Barbershops		Hairdressing/ladies salons		Unisex salons	
	No. examined	Resistant, *n* (%)	No. examined	Resistant, *n* (%)	No. examined	Resistant, *n* (%)
*S. aureus*	12	5 (41.6)	12	2 (16.6)	28	2 (7.1)
*S. epidermidis*	21	1 (4.7)	18	0 (0)	16	1 (6.25)
*S. liquefaciens*	3	3 (100)	3	3 (100)	2	2 (100)
*S. xylosus*	5	5 (100)	2	0 (0)	4	3 (75)
*E. aerogenes*	5	4 (80)	0	0 (0)	3	3 (100)
*S. marcescens*	2	2 (100)	0	0 (0)	0	0 (0)
*S. sciuri*	0	0 (0)	0	0 (0)	1	1 (100)

Total	48	20 (41.7)	35	5 (14.3)	54	12 (32)

*n*, number; %, percentage.

**Table 6:** Blast comparison between *S. aureus* disinfectant resistance protein (*qac*A) genes identified and other *Staphylococcus* spp. in Gene Bank using NCBI Blast.

No.	Organisms	Sequence	Accession number	References	Percentage identity (%)	*E* value
**1**	*S. aureus*	STP6	NA	This study	100.00	0.027
**2**	*S. aureus*	STP9	NA	This study	100.00	0.027
**3**	*S. aureus*	NSNJ5	LC335721.1	Saber et al. [32]	100.00	0.027
**4**	*S. aureus*	NSNJ4	LC335720.1	Saber et al. [32]	100.00	0.027
**5**	*S. aureus*	NSNJ3	LC335719.1	Saber et al. [32]	100.00	0.027
**6**	*S. aureus*	NSNJ2	LC335718.1	Saber et al. [32]	100.00	0.027
**7**	*S. aureus*	NSNJ1	LC335717.1	Saber et al. [32]	100.00	0.027
**8**	*S. aureus*	TPS89 pTZ2089	NG_048037.1	Nakaminami et al. [33]	100.00	0.027
**9**	*S. aureus*	Teh11	KP687798.1	Hassanzadeh et al. [34]	100.00	0.027
**10**	*S. aureus*	FJ857944.1	FJ857944.1	Zhou et al. [35]	100.00	0.027
**11**	*S. epidermidis*	MK040371.1 strain 20.1	MK040371.1	Addetia et al. [36]	100.00	0.027
**12**	*S. epidermidis*	MK040366.1 strain 106.1	MK040366.1	Addetia et al. [36]	100.00	0.027
**13**	*S. epidermidis*	MK040365.1 strain 99.1	MK040365.1	Addetia et al. [36]	100.00	0.027
**14**	*S. epidermidis*	MK040364.1 strain 68.5	MK040364.1	Addetia et al. [36]	100.00	0.027
**15**	*S. epidermidis*	MK040363.1 strain 66.3	MK040363.1	Addetia et al. [36]	100.00	0.027
**16**	*S. hominis*	MK040362.1 strain 125.1	MK040362.1	Addetia et al. [36]	100.00	0.027
**17**	*S. epidermidis*	MK040362.1 strain 36.5	MK040361.1	Addetia et al. [36]	100.00	0.027

## Data Availability

The data in tables and figures used to support the findings of this study are included in the article.
